# HNRNPH1 Is a Novel Regulator Of Cellular Proliferation and Disease Progression in Chronic Myeloid Leukemia

**DOI:** 10.3389/fonc.2021.682859

**Published:** 2021-07-06

**Authors:** Menghan Liu, Lin Yang, Xiaojun Liu, Ziyuan Nie, Xiaoyan Zhang, Yaqiong Lu, Yuxia Pan, Xingzhe Wang, Jianmin Luo

**Affiliations:** Department of Hematology, The Second Hospital of Hebei Medical University, Key Laboratory of Hematology, Shijiazhuang, China

**Keywords:** CML, HNRNPH1, PTPN6, PI3K/AKT, proliferation, apoptosis

## Abstract

RNA binding proteins act as essential modulators in cancers by regulating biological cellular processes. Heterogeneous nuclear ribonucleoprotein H1 (HNRNPH1), as a key member of the heterogeneous nuclear ribonucleoproteins family, is frequently upregulated in multiple cancer cells and involved in tumorigenesis. However, the function of HNRNPH1 in chronic myeloid leukemia (CML) remains unclear. In the present study, we revealed that HNRNPH1 expression level was upregulated in CML patients and cell lines. Moreover, the higher level of HNRNPH1 was correlated with disease progression of CML. *In vivo* and *in vitro* experiments showed that knockdown of HNRNPH1 inhibited cell proliferation and promoted cell apoptosis in CML cells. Importantly, knockdown of HNRNPH1 in CML cells enhanced sensitivity to imatinib. Mechanically, HNRNPH1 could bind to the mRNA of PTPN6 and negatively regulated its expression. PTPN6 mediated the regulation between HNRNPH1 and PI3K/AKT activation. Furthermore, the HNRNPH1–PTPN6–PI3K/AKT axis played a critical role in CML tumorigenesis and development. The present study first investigated the deregulated HNRNPH1–PTPN6–PI3K/AKT axis moderated cell growth and apoptosis in CML cells, whereby targeting this pathway may be a therapeutic CML treatment.

## Introduction

Chronic myeloid leukemia is a malignant polyclonal disease originating from hematopoietic stem cells characterized by the Ph chromosome, which is the ectopic of t (1, 2) (q34; q11). The formation of the BCR–ABL fusion gene encodes P210 protein with constitutive tyrosine kinase activity involved in proliferation, apoptosis, and other biological functions of CML cells (3). Although the tyrosine kinase inhibitors (TKIs) chemotherapy induces a high clinical response rate in the majority of CML patients, some of them still suffered from disease progression which means poor prognosis and shorter overall survival (4, 5). While the BCR–ABL fusion gene plays an important role in the initial stages of CML, some patients were suffered from the progression of CML because of the TKI intolerance or drug resistance. As we know, the etiology of disease progression is highly complex with wide heterogeneity, involving aberrantly activated pathways driven by the gene expression abnormalities (6, 7). Thus, understanding the underlying mechanisms of CML disease progression and search novel therapeutic targets is urgently needed.

RNA-binding proteins (RBPs), which could bind to mRNA or other RNAs, play a critical role in various biological cellular processes including transcription, translation, cleavage splicing and mRNA stability. Due to structural flexibility and domain polyfunctionality, the alterations or mutations of RBPs expression may be associated with tumorgenesis in a variety of human cancers, especially in hematologic malignancies (8, 9). However, few studies have focused on the role of RBPs in the initiation and progression of CML. Therefore, investigating the intricate network of RBPs and downstream mRNA may provide a strategy for understanding the mechanism and treatment in CML progression.

HNRNPH1, as an early reported RBPs, participates in RNA editing, RNA modification, and RNA stability (10). The aberrant overexpression of HNRNPH1 was seen in many cancers, such as gliomas, esophageal cancer, rhabdomyosarcoma and hepatocarcinoma (1, 11–13). Previously studies have confirmed that high expression level of HNRNPH1 could moderate tumorigenesis not only by upregulating the expression of oncogenes but also inhibiting the expression of tumor suppressor genes, such as P53, Ron and BCL-X (14–17). Furthermore, HNRNPH1 may also contribute to the drug response in gastric cancer cells (18). Importantly, a previous study has confirmed that HNRNPH1 is frequently elevated in AML patients. Knockdown of HNRNPH1 correlated to the cell proliferation in AML cells (1). However, the role of HNRNPH1 in CML has rarely been investigated.

In the present study, we found that the HNRNPH1 level was upregulated in CML patients, especially the CML progression phase. The HNRNPH1 downregulation inhibited cell proliferation, induced cell apoptosis, and arrested the cell cycle of CML cells *in vivo* and *in vitro*. Moreover, HNRNPH1 was revealed as a member of RBPs, affecting the PI3K/AKT pathway by regulating the PTPN6 expression through binding to its mRNA. The findings of this study provide a deep insight into the metabolic dysfunctions in CML progression and a novel potential therapeutic target for CML patients in the future.

## Materials and Methods

### Specimen Collection

Bone marrow mononuclear cells (BM-MNCs) were extracted from 60 newly diagnosed and untreated CML patients between 2016 and 2020 in the Department of Hematology of the Second Hospital of Hebei Medical University, Shijiazhuang, China. Full detailed information of the patient characteristics is presented in **Table 1**. Furthermore, BM-MNCs of 30 healthy donors were used as normal controls (NC). Chronic myeloid leukemia was diagnosed by molecular biology, bone marrow morphology, immunology, and cytogenetics examination (19, 20). Patients with severe cardiopulmonary, renal or liver failure or coagulation abnormality or pregnant were excluded. The BM-MNCs were extracted by lymphocyte isolation fluid following the instructions. After centrifugation, the cell layer was collected for analysis. Red blood cells were lysed using RBC lysis solution, and samples were washed twice with PBS. The ethics committee of the Second Hospital of Hebei Medical University approved this experiment.

**Table 1:** Characteristics of the patients included in the study.

Characteristic	CML-CP (n = 30)	CML-AP (n = 18)	CML-BP (n = 12)
Age (years), median (range)	51 (21–79)	49 (25–66)	57 (36–73)
Male/female (n/n)	19/11	9/9	4/8
WBC count, ×10^9^/L, median (range)	155.4 (23.5–527.5)	72.5 (1.75–433)	78.7 (1.5–327.9)
Haemoglobin level (g/l)	105 (70–147)	85 (52–118)	92 (56–124)
Platelet count, ×10^9^/l, median (range)	522 (3–1,476)	609 (4–3,000)	151 (11–576)

### Cell Culture

K562 and KCL22 cells were chosen as representatives of the CML cell lines. HL-60, THP-1, and U937 cells were acute leukemia cell lines. All cell types were maintained in our laboratory. All above cells were cultured in RPMI 1640-based culture medium (Gibco) or Iscoves-modified Dulbecco’s medium (IMDM; Gibco) culture medium supplemented with 10% fetal bovine serum (FBS; Gibco). The aforementioned conditioned media contained 100 units/ml penicillin and 100 ug/ml streptomycin. The cells were cultivated in an incubator at 37°C, 95% air and 5% CO_2_ saturated humidity (Thermo, Waltham, MA, USA).

### Cell Transfection

Lentiviruses containing shRNA-HNRNPH1 or overexpression of PTPN6 plasmid were constructed by the Shanghai Genechem Co., Ltd. (Shanghai, China). The multiplicity of infection (MOI) refers to the proportion of infectious viruses per cells. K562 cells were infected with each virus at an MOI of 30. The MOI of KCL22 was 40. The virus was added into cells from the logarithmic growth stage according to the infection conditions and cultured for 12–16 h. Then the cells were incubated with culture medium containing 10% FBS. The cells were treated with puromycin at 2 μg/ml to screen for stably transfected cells lines on conditions.

### Cell Viability Assay

The cell viability was measured using the CCK-8 (Beibo Biological Reagent Co., Shanghai, China) assay. Approximately 100 ul of mixed suspension cells (1 × 10^5^ cells/ml) required for different experiments were added to the 96-well plate. Following cell culture, 10ul CCK-8 solution was added into each plate at various time points and incubated at 37°C in 5% CO_2_ saturated humidity for 2 h. The optical density was read at 450 nm in a microplate reader (BioTek, Winooski, VT, USA) in different time points.

### Cell Apoptosis Assay

Cell apoptosis was assayed by an Annexin V/FITC/PI Apoptosis Detection Kit (BD Biosciences, Franklin Lakes, NJ, USA). The cells required for different experiments were mixed with 5 µl Annexin V/FITC and 10 µl propidium iodide (PI) based on the manufacturer’s instructions. They were analyzed with a FC500 flow cytometer (Beckman Coulter). The data was performed using Kaluza software (Beckman Coulter).

### RNA Extraction and Quantitative Real-Time PCR

qRT-PCR analysis was performed with standard procedure as described previously (21). Briefly, total RNA of cells lines and BM-MNCs were extracted using Trizol (Invitrogen, Carlsad, CA, USA). The cDNA was synthesized by a SureScriptTM First-Strand cDNA Synthesis Kit (Funeng, Guangzhou, China). Quantitative real-time reverse transcriptase-polymerase chain reaction (qRT-PCR) was performed by an All-in-OneTM qPCR Mix (Funeng, Guangzhou, China). Each reaction was dependently repeated thrice. qRT-PCR was performed at 95°C for 10 min, followed by 40 cycles of 95°C (15 s), 60°C (30 s), and 72°C (30 s). Moreover, GAPDH was used as an internal reference.

### Western Blot Analysis

Proteins were obtained using radioimmunoprecipitation assay (RIPA) buffer to dissolve the cells. The quantification was tested using bicinchoninic acid Protein Assay Kit (Boster Biological Company, Ltd., Wuhan, China). All protein samples were subjected to 10% sodium dodecyl sulfate-polyacrylamide gel electrophoresis. The target strip was transferred to a polyvinylidene fluoride (PVDF) membrane (Millipore, Burlington, MA, USA) *via* electrophoresis. The PVDF membrane was blocked in 5% nonfat milk. The protein bands were incubated with specific primary antibodies as follows: HNRNPH1 (1:2,000) (ab 154894, Abcam, CA, USA), PTPN6 (1:1,000) (ab 32559, Abcam, CA, USA), AKT (1:1,000) (ab 38449, Abcam, CA, USA), p-AKT (1:1,000) (ab 8805, Abcam, CA, USA), and β-ACTIN (1:8,000; Abways Technology, New York, NY, USA; AB0035). Moreover, the PVDF membrane was incubated in a goat-anti-rabbit secondary antibody (1:10,000, Boster Biological Company, Ltd., Wuhan, China) overnight. Images of protein quantification were captured by BioSpectrum Imaging System (UVP, LLC, Upland, CA, USA).

### Immunofluorescence Staining

Cell samples were centrifuged, fixed, and smeared onto coverslips. The cells were fixed using 4% formaldehyde and preincubated with 10% normal goat serum (710,027, KPL, Gaithersburg, MD, USA). The cells were rinsed with PBS and incubated with anti-HNRNPH1 antibody (Abcam, ab 154894) at 37°C for 1 h. Immunofluorescence staining was enhanced using a rabbit anti-red fluorescent-labeled antibody (Rockland Immunochemicals Inc., Gilbertsville, PA, USA; 1:100). The smears were then incubated with DAPI. Furthermore, nuclear staining was observed by confocal microscopy (Zeiss LSM 700, Germany) and digitized with confocal software.

### Colony-Forming Assay

The colony formation was performed with standard procedure in accordance with the method described in previous study (2, 22). The logarithmic growth phase of treated cells was added to the 1% methylcellulose for 14–18 days at 37°C with 5% CO_2_ saturated humidity. The methylcellulose medium was mixed with powder culture medium with ultrapure water, 40% FBS, and 1% penicillin/streptomycin. The colonies were quantified as aggregates with greater than 50 cells by microscope (Axio-observer D1, Zeiss, Germany). Then the colonies were counted manually and photographed.

### RNA Immunoprecipitation

The cells were treated with cell lysis buffer. The 10% lysis sample (named input) was stored, and 80% (named IP) was used in immunoprecipitation reactions with HNRNPH1 antibody (Abcam No 154894), and 10% (named IgG) was incubated with rabbit IgG (Cell Signaling Technology, Danvers, MA, USA) as a negative control The RNA of input and IP was extracted using TRIzol reagent (Invitrogen). The purified RNA samples were analyzed with conventional RT-PCR.

### RNA-Seq

Three biological replicates in each control and knockdown HNRNPH1 groups in K562 cells were collected for microarray analysis after 14 days of infection. The total RNA was extracted using TRIzol reagent (Invitrogen) and subject to RNA sequencing. RNA seq was performed by BGI Technology Services Co., Ltd (Shenzhen, China). The differentially expressed genes were screened based on fold change (>0.5) and Student’s t-test (P <0.05). The RNA-sequencing raw data have been deposited into sequence read archive (SRA) database (https://www.ncbi.nlm.nih.gov/sra).

### Animal Experiment

Sixteen female severe combined immunodeficient (SCID) athymic nude mice (16–20 g, 5–6 weeks’ old) were purchased from SPF Biotechnology Co., Ltd. (Beijing, China). All mice were raised at an ambient temperature of 18–22°C and relative humidity of 50–60%. The stably knockdown-HNRNPH1 K562 cells and negative control K562 cells were subcutaneously injected into the left dorsal flanks of each mouse (0.5 × 10^6^ cells per injection). Tumor size and body weight were dynamically observed. The nude mice were sacrificed on day 28 post-inoculation. All animal experiment protocols were approved by the committee on animal experimentation of Hebei Medical University and carried out following the guidelines on animal experimentation.

### Immunohistochemistry

The tumor tissues of mice were fixed with 4% paraformaldehyde and routinely dehydrated, embedded in paraffin sections, and cut into 4-μm thick sections. The antigen repair was then performed with sodium citrate. Moreover, endogenous peroxidase was inactivated by 3% hydrogen peroxide. Tissue sections were incubated with the primary antibodies at 4°C overnight and with secondary antibody at room temperature for 30 min respectively. Sections were incubated with DAB chromogen at room temperature for 3–10 min after washing with phosphate-buffered saline with detergent Tween. Slices were sealed with coverslips after rinsing with running water and hematoxylin counterstain.

### Statistical Analysis

The data were presented by means ± SD. Student’s t-test and Chi-square test were applied to detect the significant difference by using the Statistical Package for the Social Sciences, version 13.0 (SPSS Inc., Chicago, IL, USA). P values <0.05 were considered statistically significant.

## Results

### HNRNPH1 Is Upregulated in CML Patients and Cell Lines

**Figure 1:**
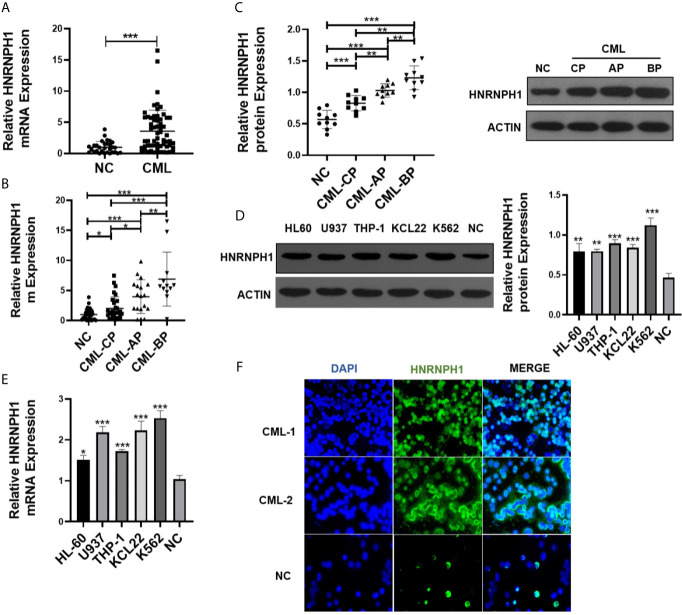
HNRNPH1 is upregulated in CML patients and cell lines. **(A)** qRT-PCR was used to detect HNRNPH1 mRNA level in BM-MNCs of CML patients and BM-MNCs of healthy donors. Normalized to GAPDH. ***P < 0.001 *vs*. NC. **(B)** qRT-PCR was used to detect HNRNPH1 mRNA level in different phases of CML (CML-CP, CML-AP, and CML-BP) patients’ BM-MNCs compared with normal controls. *P < 0.05, **P < 0.01, ***P < 0.001. **(C)** Western blot was used to detect HNRNPH1 protein levels in different phases of CML (CML-CP, CML-AP, and CML-BP) patients’ BM-MNCs compared with normal controls. Left panel, scatter plots of protein densitometric analysis. **P < 0.01, ***P < 0.001. **(D)** Western blot was used to detect HNRNPH1 protein levels in leukemia cell lines (HL-60, U937, THP-1, K562, and KCL22) and BM-MNCs of normal controls. Right panel, densitometric analysis. **P < 0.01, ***P < 0.001 *vs*. NC. **(E)** qRT-PCR was used to detect HNRNPH1 mRNA levels in leukemia cell lines (HL-60, U937, THP-1, K562, and KCL22) and BM-MNCs of normal controls. *P < 0.05, ***P < 0.001 *vs.* NC. **(F)** Immunofluorescence analyzed the HNRNPH1 protein level and localization of HNRNPH1 in PBMCs of CML-CP patients and BM-MNCs of normal controls.

The difference in the HNRNPH1 expression between the BM-MNCs of CML patients and the healthy donors was first explored to investigate the role of HNRNPH1 in CML. qRT-PCR result showed that mRNA expression of HNRNPH1 in BM-MNCs of CML patients was significantly higher compared with the normal controls (**Figure 1A**). The HNRNPH1 expression in different progressions of CML was then compared. Moreover, the HNRNPH1 expression in the progressive phase was higher compared with the chronic phase (**Figure 1B**). However, the expression level in the blast phase was the highest. HNRNPH1 protein level was then also detected in the BM-MNCs of 10 patients selected in different stages of CML and healthy donors. The Western blot analysis result showed that the HNRNPH1 protein level was significantly increased in CML patients compared with normal controls. Consistent with qRT-PCR results, the HNRNPH1 protein level was increased in the blast and accelerated phase compared with the chronic phase (**Figure 1C**). Furthermore, mRNA and HNRNPH1 protein levels were found to be upregulated in leukemia cell lines compared with BM-MNCs of healthy donors (**Figures 1D, E** **)**. Immunofluorescence also displayed that HNRNPH1 protein was primarily localized in the nucleus and increased in CML patients (**Figure 1F**). These results suggest that HNRNPH1 expression is abnormally elevated in CML, especially in the progressive phase.

### HNRNPH1 Downregulation Promotes Apoptosis of CML Cells

**Figure 2:**
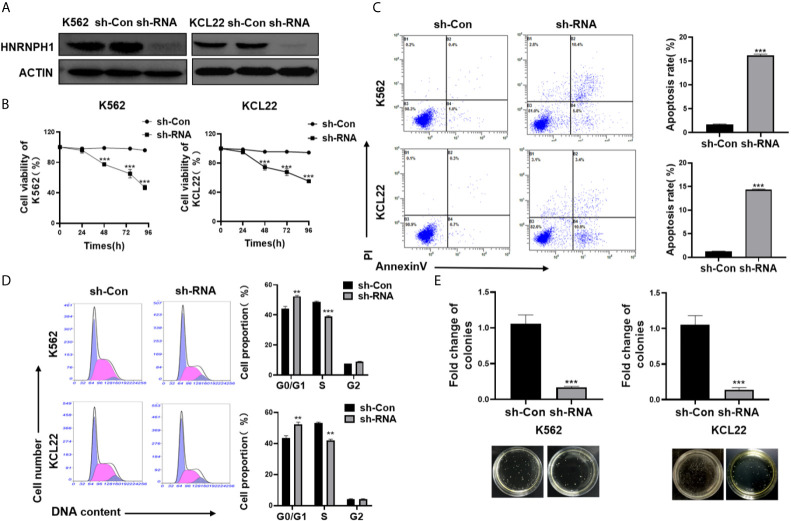
HNRNPH1 downregulation promotes apoptosis of CML cells. **(A)** K562 and KCL22 cells were transfected with specific sh-RNA of HNRNPH1 or negative sh-RNA (sh-Con). Western blot was used to detect HNRNPH1 protein level. **(B)** CML cells were prepared as **(A)**, CCK-8 analysis was used to detect cell proliferation. ***P < 0.001 *vs.* sh-Con. **P < 0.01 *vs.* sh-Con. **(C)** CML cells were prepared as **(A)**, and cell apoptosis rate was detected by flow cytometry using Annexin V-FITC/PI staining. The right panel shows the apoptosis rate from three independent experiments. ***P < 0.001 *vs.* sh-Con. **(D)** CML cells were prepared as **(A)**, and the cell cycle was detected by flow cytometry. The right panel shows the cell proportion of three independent experiments. **P < 0.01 *vs.* sh-Con. **(E)** CML cells were prepared as **(A)**, and cell proliferation was detected by colony formation assay. ***P < 0.001 *vs.* sh-Con. The bottom panel shows an image of colonies.

HNRNPH1 was knocked by transfection of sh-RNA or empty vector (sh-Con) into CML cell lines to investigate the impact of HNRNPH1 on CML progression. The sh-RNA transfection of HNRNPH1 successfully reduced the HNRNPH1 expression level in both K562 and KCL22 cells compared with the negative controls (**Figure 2A**). Cell viabilities were then measured by CCK-8 assay. The HNRNPH1 downregulation markedly reduced cell proliferation compared with negative controls (**Figure 2B**). The cell apoptosis by flow cytometry using Annexin V-FITC/PI staining was then detected next. The result showed that HNRNPH1 knockdown in CML cells promoted cell apoptosis compared with the negative controls (**Figure 2C**). Consistently, HNRNPH1 suppression was found to inhibit the multiplication of CML cells by mainly arresting their cell cycle at G1/G0 phase(K562 44.34% *vs.* 52.35%, KCL22 43.61% *vs.* 53.60%)and caused S phase reduction(K562 48.58% *vs.* 38.86%, KCL22 52.41% *vs.* 41.9%), suggesting that HNRNPH1 knockdown played an inhibitory effect on cell arrest phase (**Figure 2D**). Furthermore, colony formation experiments also confirmed that HNRNPH1 knockdown inhibited cell proliferation (**Figure 2E**). Taken together, these results revealed that HNRNPH1 played a curtail role in CML cell survival *in vitro*.

### HNRNPH1 Knockdown Increases the Sensitivity of Imatinib in CML Cells

**Figure 3:**
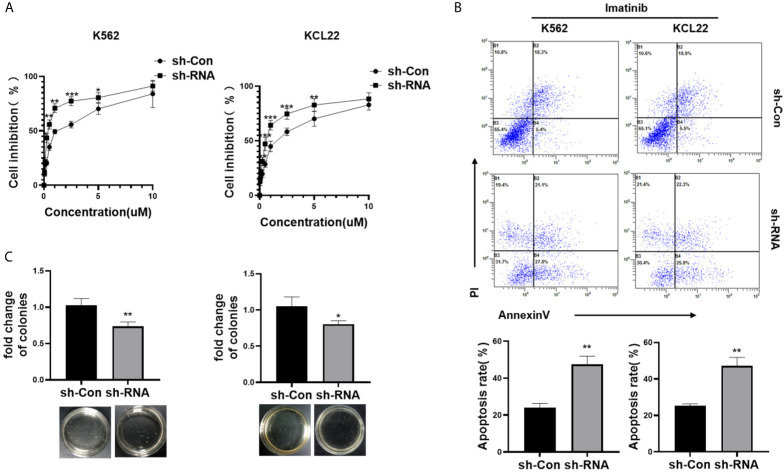
HNRNPH1 knockdown increases imatinib sensitivity in CML cells. **(A)** K562 and KCL22 cells were transfected with specific sh-RNA of HNRNPH1 or negative sh-RNA (sh-Con) and treated with different imatinib concentrations for 48 h. CCK-8 analysis was used to detect cell inhibition. ***P < 0.001, **P < 0.01 *vs*. sh-Con. **(B)** K562 and KCL22 cells were transfected with specific sh-RNA of HNRNPH1 or negative sh-RNA (sh-Con) and treated with imatinib (3 µM) for 48 h. Cell apoptosis was detected by flow cytometry using Annexin V/FITC/PI. The right panel shows the apoptosis rate from three independent experiments. **P < 0.01 *vs*. sh-Con. **(C)** CML cells were prepared as **(B)**, and cell proliferation was detected by colony formation assay. *P < 0.05, **P < 0.01 *vs*. sh-Con. The bottom panel shows an image of colonies.

CML cells were transfected with sh-RNA of HNRNPH1 or sh-Con and then treated with different concentrations of imatinib to investigate the effect of HNRNPH1 on imatinib sensitivity. The IC50 of imatinib was detected by the CCK-8 assay. Moreover, HNRNPH1 silencing significantly increased imatinib sensitivity compared with the negative control (**Figure 3A**). Consequently, the number of apoptotic cells induced by imatinib was increased in the knockdown group compared with the negative control (**Figure 3B**). Moreover, colony formation assay showed that HNRNPH1 knockdown in CML cells could promote cell sensibility to imatinib (**Figure 3C**). These results suggest that HNRNPH1 knockdown promotes imatinib sensitivity in CML cells.

### HNRNPH1 Regulates the PTPN6 Expression

**Figure 4:**
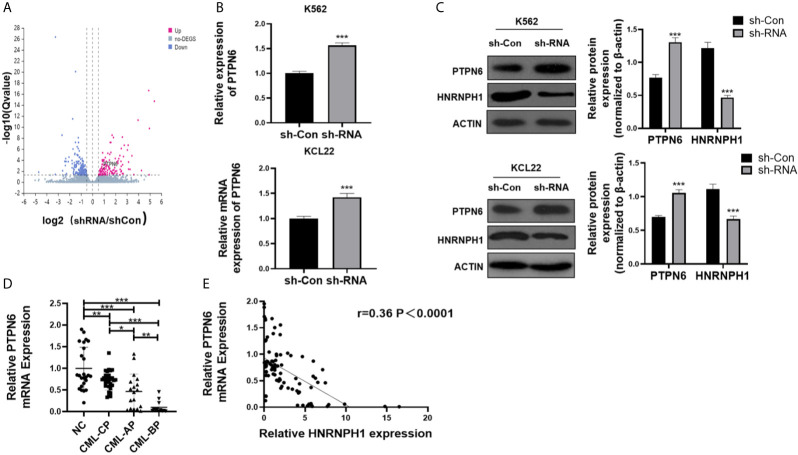
HNRNPH1 regulates the PTPN6 expression. **(A)** Three independent HNRNPH1 reduced and control cells were prepared for RNA preparation and microarray analysis. Genes with altered expression were displayed in volcano plots. The upregulated genes are highlighted in pink and downregulated genes in blue. **(B)** K562 and KCL22 cells were transfected with specific sh-RNA of HNRNPH1 or negative sh-RNA (sh-Con). qRT-PCR was used to detect the PTPN6 mRNA level. ***P < 0.001 *vs.* sh-Con. **(C)** CML cells were prepared as **(B)**, western blot was used to detect PTPN6 protein level. Right panel, densitometric analysis. ***P < 0.001 *vs.* sh-Con. **(D)** qRT-PCR was used to detect PTPN6 mRNA level in different phases of CML (CML-CP, CML-AP, and CML-BP) patients’ BM-MNCs compared with normal controls. *P < 0.05, **P < 0.01, ***P < 0.001. **(E)** Pearson correlation analysis was used to analyze the relationship between HNRNPH1 and PTPN6 in BM-MNCs of CML patients (R = 0.36, P < 0.0001).

HNRNPH1 was knocked down in K562 cells to identify molecular mechanisms of HNRNPH1 in regulating the growth of CML cells. RNA-seq was then performed. In addition, the differential expressed genes were displayed in the volcano plot between the sh-RNA and negative control groups (**Figure 4A**). Among those relative upregulated genes, PTPN6, which had been confirmed to play a crucial role as a cancer suppressor in leukemia, was focused on (21). The HNRNPH1 was knocked down in K562 cells to confirm the results of the RNA-seq and detect the PTPN6 expression. Consistent with the result of RNA-seq, the fold change of PTPN6 expression was upregulated in HNRNPH1 knocked down cells than control cells by qRT-PCR analysis. As shown in **Figure 4B**, the fold change of PTPN6 mRNA expression in K562 and KCL22 cell were 1.56 and 1.42, respectively. Likewise, the western blot result showed that the protein level of PTPN6 was increased in HNRNPH1-reduced cells compared with the negative control (**Figure 4C**). Additionally, the PTPN6 expression in CML patients was detected by qRT-PCR analysis. The PTPN6 expression which was reduced in CML-CP patients compared with healthy donors was related to illness aggravation (**Figure 4D**). A significant inverse correlation existed between the HNRNPH1 expression and PTPN6 in CML patients (**Figure 4E**). Taken together, these data revealed that PTPN6, which was downregulated in CML, may negatively correlate with HNRNPH1.

### HNRNPH1 Regulates PI3K/AKT Pathway by Binding to PTPN6

**Figure 5:**
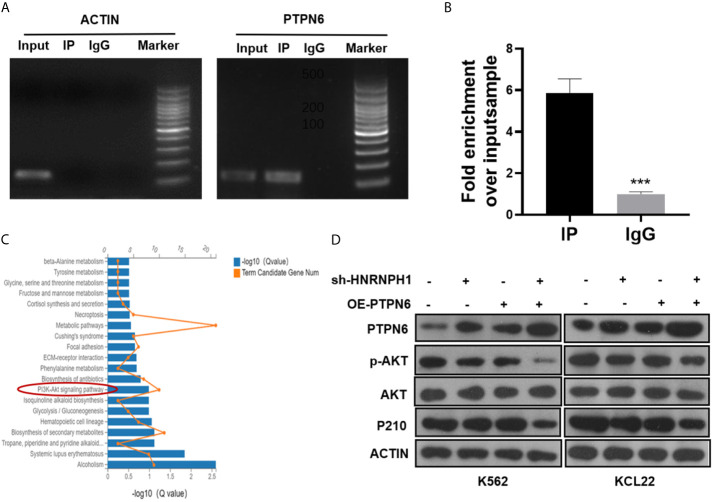
HNRNPH1 regulates PI3K/AKT pathway by binding to PTPN6. **(A, B)** RIP-PCR and agarose gel electrophoresis were used to test the interaction between the HNRNPH1 protein and PTPN6 mRNA. ***P < 0.001 *vs.* IgG **(C)**. KEGG pathway significant enrichment was used to identify the main biochemical metabolic and signal transduction pathways involved in differentially expressed genes. **(D)** CML cells were transfected with sh-HNRNPH1 or PTPN6 overexpression plasmid or co-transfected them together. Western blot analysis was used to detect PTPN6, p-AKT, AKT, and P210BCR-ABL protein levels.

As an RNA binding protein, HNRNPH1 is involved in gene regulation by directly assembling on its mRNA (23). Radioimmunoprecipitation (RIP) followed by RT-PCR and qRT-PCR were performed to investigate whether HNRNPH1 could bind to the mRNA of PTPN6 to regulate its expression. RIP-PCR analysis showed that PTPN6 mRNA, but not Actin, was present in the protein–RNA complex pulled down by the antibody HNRNPH1 (**Figures 5A, B** **)**, indicating that HNRNPH1 could physically bind to the mRNA of PTPN6. The screened differential genes were enriched in the PI3K/AKT pathway (**Figure 5C**), just like a previous study reported that PTPN6 could influence the activity of the PI3K/AKT pathway (24). To further verify whether HNRNPH1 contributed to the regulation of the PI3K/AKT pathway, CML cells were transfected with sh-HNRNPH1 or PTPN6 overexpression plasmid or co-transfected them together. The western blot results showed that PTPN6 overexpression could decrease the p-AKT protein level, while this reduction effect of p-AKT could be further reduced by HNRNPH1 knockdown together in CML cells (**Figure 5D**), suggesting that PTPN6 may mediate the relationship between the HNRNPH1 and the activation of PI3K/AKT in CML cells. Interestingly, this study also found that the expression trend of P210 protein that encoded BCR–ABL1 fusion gene was surprisingly consistent which is regulated positively by HNRNPH1 but negatively by PTPN6. Collectively, these results revealed that HNRNPH1 contributed to CML cell progression by moderating PI3K/AKT pathway.

### HNRNPH1 Downregulation Inhibits CML Cell Growth *In Vivo*

**Figure 6:**
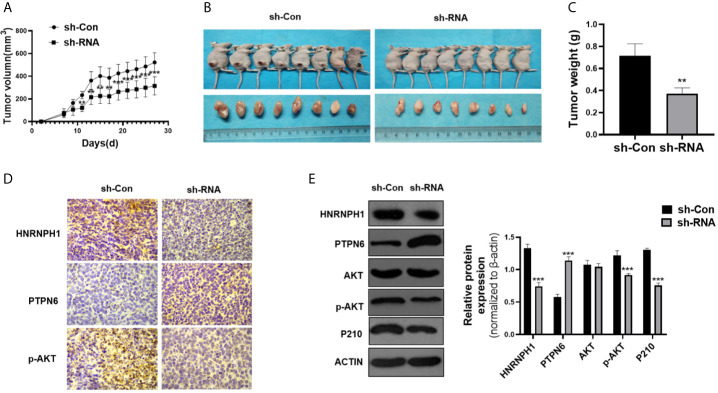
HNRNPH1 downregulation inhibits CML cell growth *in vivo*. **(A)** K562 cells were engineered to stably knockdown HNRNPH1 and the cells were then subcutaneously injected into the nude mice to establish CML xenograft tumors. Tumor volumes were monitored by direct measurement. **P < 0.01, ***P < 0.001 *vs.* sh-Con. **(B)** Representative tumor sizes of xenograft mice in each group. **(C)** The xenograft tumor wet weight in each group of mice. **P < 0.01 *vs.* sh-Con. **(D)** Immunohistochemistry stain was used to measure the HNRNPH1, PTPN6, and P-AKT protein levels in xenograft tumors. **(E)** Western blot was used to detect the PTPN6, p-AKT, AKT, and P210BCR-ABL protein levels in xenograft tumors. Right panel, densitometric analysis. ***P < 0.001 *vs.* sh-Con.

The nude mice xenograft model was applied to confirm whether the reduction of HNRNPH1 expression could inhibit the proliferation of CML cells through the PTPN6–PI3K/AKT pathway *in vivo*. The shRNA K562 cell lines stably suppressing HNRNPH1 were screened. The HNRNPH1-reduced K562 and control cells were then subcutaneously implanted into the nude mice. The HNRNPH1 reduction could inhibit the tumor size compared with the control *in vivo* (**Figures 6A, B** **)**. Similarly, this study also found that downregulating the HNRNPH1 expression could decrease the weight of xenograft tumors (**Figure 6C**). In addition, the protein level of HNRNPH1, PTPN6, AKT, p-AKT and BCR-ABL was detected by immunohistochemical (**Figure 6D**) and western blot analysis (**Figure 6E**). The results showed that the PTPN6 protein level increased while the p-AKT and BCR-ABL dramatically decreased in the HNRNPH1 reduction group compared with the negative control. The results proved that HNRNPH1 reduction also inhibited CML cell growth *in vivo* through the PTPN6-PI3K/AKT pathway.

## Discussion

The pathogenesis of CML is a complex progress that is concerned with numerous mechanisms. Therefore, many studies have been dedicated to elucidate the pathogenesis of advanced CML. Our previous studies have shed light on underlying mechanisms linked to disease progression, such as epigenetic alterations, abnormal activation of coding or non-coding RNA transcripts and cancer-related pathways. For example, the PTPN6 expression is closely related to the progression of CML, which is regulated by epigenetics modifications including acetylation and methylation (21). The Long Noncoding RNA MEG3 which could sponge to miRNA inhibit the CML cell proliferation in CML cells and contributes to CML progression (25, 26). Moreover, the deubiquitinating enzyme ubiquitin-specific peptidase 15 expression level was significantly downregulated in CML patients by blocking JAK/STAT5 pathway and involved in imatinib resistance (27). However, the specific mechanisms of CML development and progression remain exclusive.

Growing evidence have proved that the post-transcription is a critical mechanism for regulating gene expression. RBPs are considered as essential modulators in RNA translation, transcription, splicing and mRNA stability, which are frequently dysregulated in tumor cells (28, 29). An increasing number of studies have indicated the critical role of RBP in hematological malignancies. For example, Wang et al. have reported that degradation of RBM39 sustains leukemia survival by altering the splicing of HOXA in AML cells (8). Gallardo et al. found that the upregulation of hnRNPK regulates MYC expression in B-cell lymphoma post-transcriptionally and translationally (9). Ge revealed that RBM25 has an impact on the AML development as a splicing factor of c-myc (30). In the present study, we found thatHNRNPH1 was upregulated in CML patients and correlated to disease progression. HNRNPH1 could function as a diagnosis related biomarker and a novel target for combination therapy for CML patients. Due to the limitations, a single-center study with a low number of patients was afforded, where subsequent research is needed to delineate the relationship with the prognosis.

Currently, some hnRNPs have been demonstrated to be involved in the oncogenesis of human hematologic malignancies. For instance, HNRNPK was downregulated in leukemia cells. Knockdown of HNRNPK promoted tumor growth of myeloproliferative neoplasm *in vivo* (31). HNRNPD also had an effect on the proliferation of BCR-ABL-positive cell lines which were increased in hematopoietic stem cells (32). The alternative splice alterations of HNRNPA2B1 influenced the development and adverse outcome in myelodysplastic syndromes (33). Despite having some HNRNPs family members being confirmed to function as oncogenes or tumor suppressor genes in CML (32, 34–36), a few of them have clearly revealed the molecular mechanisms in CML progression. HNRNPH1, which is localized in the nucleus, has been shown aberrantly overexpressed in hematological malignancies including AML, Burkitt lymphoma, and T-acute lymphoblastic leukemia (8, 37, 38). Importantly, Yamazaki et al. also found that HNRNPH1 plays a crucial role in stem cell maintenance and hematopoietic development suggesting that HNRNPH1 may be involved in the regulation of hematopoietic stem cells (39). In the present study, we demonstrated that HNRNPH1 expression was higher in CML patients compared with healthy donors, and gradually elevated along with disease progression that blast crisis patients have significantly highly expressed HNRNPH1 (**Figures 1A–C**). The higher level of HNRNPH1 has also been observed in leukemia cell lines and was particularly prominent in CML cells. Given that some patients in the progressive phases are associated with TKIs insensitive or resistance, HNRNPH1 downregulation was found to increase imatinib sensitivity. The above results potentially indicated that HNRNPH1 was an important molecular marker for CML progression and may help improve treatment, such as early turn to more efficacious TKIs or combination chemotherapy. Our data further elaborated that the reduction of HNRNPH1 expression can promote apoptosis and inhibit proliferation in CML cells. Therefore, HNRNPH1 was shown to be an important regulator for the proliferation of CML cells. These findings further validated that HNRNPH1 may serve as therapeutic target for CML treatment as its functions as the anti-apoptotic molecule in tumor metastasis and growth. Notably, whether the downregulation of HNRNPH1 would arrest or even reverse disease progression still requires further investigations.

Besides the potential use of HNRNPH1 as a biomarker of CML disease progression, underlying mechanisms of HNRNPH1 were identified. PTPN6, possessing an SH2 domain, was a tumor suppressor by dephosphorylation in CML (21, 40). RIP-PCR revealed that HNRNPH1 could be a negative upstream regulator by regulating the mRNA expression of PTPN6 directly. Gratifyingly, HNRNPH1 motif sequence was found to be present in the PTPN6 gene, suggesting that HNRNPH1 regulated the post-transcription level of PTPN6, which supported the experimental results in our study (41). However, our experiments did not fully address the mechanism whether HNRNPH1 affects the stability or splicing of PTPN6, which still needs to be further explored. The tyrosine kinase activity of BCR-ABL activate multiple signaling pathways, including PI3K/AKT, of which is responsible for cell survival (42). It is consistent with previous studies that PTPN6 may inhibit the activation of PT3K/AKT pathway by mediating AKT dephosphorylation (43). The present study also made a promising discovery that BCR-ABL was positively regulated by HNRNPH1 downregulation mediated by PTPN6. A prior study demonstrated that PTPN6 was a binding protein of P210 BCR-ABL, which may explain the reason for this phenomenon (44). We have here provided the first piece of evidence that the HNRNPH1–PTPN6–PI3K/AKT axis mediated CML progression.

In this study, we demonstrated that downregulation of HNRNPH1 inhibits the potential tumorigenic both *in vitro* and *in vivo*, and can increase imatinib sensitivity, providing a rational drug design to prevent the expression of HNRNPH1 in BCR-ABL positive leukemia. It was confirmed that HNRNPH1 was indeed associated with PARylation with a conserved domain of pADPr-binding (45). Given that the reduction of PARG activity can significant elevate the PARylation cellular level (46), implying that PARG inhibitors may block the HNRNPH1 related tumor growth. We hypothesize that PARG inhibitor can serve as a targeted HNRNPH1 inhibitor for a combination of chemotherapies in CML patients with high level of HNRNPH1, which deserves further exploration.

## Conclusion

**Figure 7:**
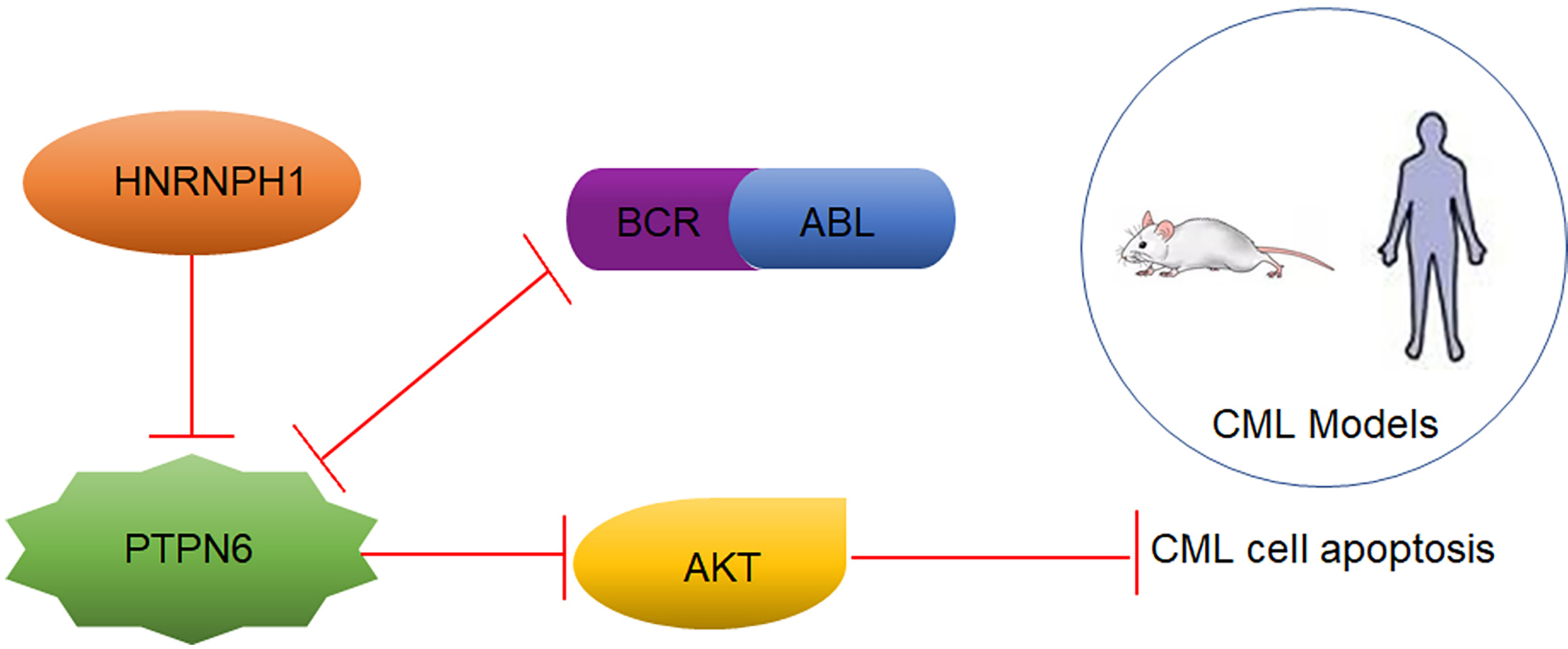
A schematic model depicting the role of HNRNPH1–PTPN6–PI3K/AKT axis in CML.

Taken together, as displayed in **Figure 7**, HNRNPH1 was uncovered, for the first time, as a potential molecular marker in CML disease progression. In addition, HNRNPH1 was also first revealed as an upstream PTPN6 regulator that can directly bind to the PTPN6 transcript. Due to the RBP nature of HNRNPH1, which has oncogene function and multiple transcriptional factor binding sites, HNRNPH1 could be a novel CML therapeutic target that has a huge potential clinical translation value. Thus, the HNRNPH1–PTPN6–PI3K/AKT axis played an important role in the genesis and CML progression.

## Data Availability Statement

The original contributions presented in the study are included in the article/supplementary material. Further inquiries can be directed to the corresponding author.

## Ethics Statement

The studies involving human participants were reviewed and approved by The second hospital of Hebei Medical University. The patients/participants provided their written informed consent to participate in this study. The animal study was reviewed and approved by Hebei Medical University.

## Author Contributions

ML, LY, and JL carry out the design of research study. XL and ZN contributed to solve the experimental problems. XZ and YL carry out the patient collection. XW and YP contributed to animal models. ML LY, and JL carry out acquisition and analysis of the data. ML wrote the manuscript. ML, LY, XL, and JL carry out revision of the manuscript. All authors contributed to the article and approved the submitted version.

## Funding

This study was partially supported by the he Natural Science Foundation of Hebei Province (H2020206438).

## Conflict of Interest

The authors declare that the research was conducted in the absence of any commercial or financial relationships that could be construed as a potential conflict of interest.

## Abbreviations

CML, chronic myeloid leukemia; RBP, RNA binding protein; HNRNPH1, Heterogeneous nuclear ribonucleoprotein H1; TKI, tyrosine kinase inhibitors; AML, acute myeloid leukemia; BM-MNCs, Bone marrow mononuclear cells; NC, normal control; FBS, Fetal bovine serum; RIP, RNA immunoprecipitation; qRT-PCR, quantitative real-time reverse transcriptase-polymerase chain reaction.

## References

[1] Lefave CV Squatrito M Vorlova S Rocco GL Brennan CW Holland EC. Splicing Factor hnRNPH Drives an Oncogenic Splicing Switch in Gliomas. EMBO J (2011) 30(19):4084–97. 10.1038/emboj.2011.259 PMC3209773 21915099

[2] Pikman Y Puissant A Alexe G Furman A Chen LM Frumm SM. Targeting MTHFD2 in Acute Myeloid Leukemia. J Exp Med (2016) 213(7):1285–306. 10.1084/jem.20151574 PMC4925018 27325891

[3] Lugo TG Pendergast AM Muller AJ Witte ON. Tyrosine Kinase Activity and Transformation Potency of Bcr-Abl Oncogene Products. Science (1990) 247(4946):1079–82. 10.1126/science.240814910.1126/science.2408149 2408149

[4] Sherbenou DW Druker BJ. Applying the Discovery of the Philadelphia Chromosome. J Clin Invest (2007) 117(8):2067–74. 10.1172/JCI31988 PMC1934568 17671641

[5] Sloma I Jiang X Eaves AC Eaves CJ. Insights Into the Stem Cells of Chronic Myeloid Leukemia. Leukemia (2010) 24(11):1823–33. 10.1038/leu.2010.159 20861912

[6] Chereda B Melo JV. Natural Course and Biology of CML. Ann Hematol (2015) 94(Suppl 20):S107–21. 10.1007/s00277-015-2325-z 25814077

[7] Modi H McDonald T Chu S Yee JK Forman SJ Bhatia R. Role of BCR/ABL Gene-Expression Levels in Determining the Phenotype and Imatinib Sensitivity of Transformed Human Hematopoietic Cells. Blood (2007) 109(12):5411–21. 10.1182/blood-2006-06-032490 PMC1890842 17347407

[8] Wang E Lu SX Pastore A Chen X Imig J Chun-Wei Lee S. Targeting an RNA-Binding Protein Network in Acute Myeloid Leukemia. Cancer Cell (2019) Mar 18 35(3):369–384.e7. 10.1016/j.ccell.2019.01.010 PMC6424627 30799057

[9] Gallardo M Malaney P Aitken MJL Zhang X Link TM Shah V. Uncovering the Role of RNA-Binding Protein Hnrnp K in B-Cell Lymphomas. J Natl Cancer Inst (2020) 112(1):95–106. 10.1093/jnci/djz078 31077320 PMC7489062

[10] Han SP Tang YH Smith R Biochem J. Functional Diversity of the hnRNPs: Past, Present and Perspectives. Biochem J (2010) 430: (3):379–92. 10.1042/BJ20100396 20795951

[11] Li Y Bakke J Finkelstein D Zeng H Wu J Chen T. HNRNPH1 Is Required for Rhabdomyosarcoma Cell Growth and Survival. Oncogenesis (2018) 7(1):9. 10.1038/s41389-017-0024-4 29362363 PMC5833419

[12] Xu H Dong X Chen Y Wang X. Serum Exosomal hnRNPH1 mRNA as a Novel Marker for Hepatocellular Carcinoma. Clin Chem Lab Med (2018) 56(3):479–84. 10.1515/cclm-2017-0327 29252188

[13] Sun YL Liu F Liu F Zhao XH. Protein and Gene Expression Characteristics of Heterogeneous Nuclear Ribonucleoprotein H1 in Esophageal Squamous Cell Carcinoma. World J Gastroenterol (2016) 22(32):7322–31. 10.3748/wjg.v22.i32.7322 PMC4997634 27621578

[14] Garneau D Revil T Fisette JF Chabot B. Heterogeneous Nuclear Ribonucleoprotein F/H Proteins Modulate the Alternative Splicing of the Apoptotic Mediator Bcl-X. J Biol Chem (2005) 280(24):22641–50. 10.1074/jbc.M501070200 15837790

[15] Decorsière A Cayrel A Vagner S Millevoi S. Essential Role for the Interaction Between Hnrnp H/F and a G Quadruplex in Maintaining p53 pre-mRNA 3′-End Processing and Function During DNA Damage. Genes Dev (2011) 25(3):220–5. 10.1101/gad.607011 PMC3034896 21289067

[16] Braun S Enculescu M Setty ST Cortés-López M de Almeida BP Sutandy FXR. Decoding a Cancer-Relevant Splicing Decision in the RON Proto-Oncogene Using High-Throughput Mutagenesis. Nat Commun (2018) 9(1):3315. 10.1038/s41467-018-05748-7 30120239 PMC6098099

[17] Panelli D Lorusso FP Papa F Panelli P Stella A Caputi M. The Mechanism of Alternative Splicing of the X-Linked NDUFB11 Gene of the Respiratory Chain Complex I, Impact of Rotenone Treatment in Neuroblastoma Cells. Biochim Biophys Acta (2013) 1829(2):211–8. 10.1016/j.bbagrm.2012.12.001 23246602

[18] Zong L Hattori N Yasukawa Y Kimura K Mori A Seto Y. LINC00162 Confers Sensitivity to 5-Aza-2’-Deoxycytidine Via Modulation of an RNA Splicing Protein, HNRNPH1. Oncogene (2019) 38(26):5281–93. 10.1038/s41388-019-0792-8 30914798

[19] Quintás-Cardama A Cortes J. Molecular Biology of bcr-abl1-positive Chronic Myeloid Leukemia. Blood (2009) 113(8):1619–30. 10.1182/blood-2008-03-144790 PMC3952549 18827185

[20] Michael W Neil P Jessica K Ellin B Ravi B Bhavana B. Chronic Myeloid Leukemia, Version 2.2021, NCCN Clinical Practice Guidelines in Oncology. J Natl Compr Canc Netw (2020) 18(10):1385–415. 10.6004/jnccn.2020.0047 33022644

[21] Zhang X Yang L Liu X Nie Z Wang X Pan Y. Haematology, Research on the Epigenetic Regulation Mechanism of the PTPN6 Gene in Advanced Chronic Myeloid Leukaemia. Br J Haematol (2017) 178(5):728–38. 10.1111/bjh.14739 28480959

[22] Wang LX Wang JD Chen JJ Long B Liu LL Tu XX. Aurora A Kinase Inhibitor Aki603 Induces Cellular Senescence in Chronic Myeloid Leukemia Cells Harboring T315i Mutation. Sci Rep (2016) 6:35533. 10.1038/srep35533 27824120 PMC5099696

[23] Neckles C Boer RE Aboreden N Cross AM Walker RL Kim B. HNRNPH1-Dependent Splicing of a Fusion Oncogene Reveals a Targetable RNA G-Quadruplex Interaction. RNA (2019) 25(12):1731–50. 10.1261/rna.072454.11920 PMC6859848 31511320

[24] Wang Y Zhu Z Church TD Lugogo NL Que LG Francisco D. SHP-1 as a Critical Regulator of Mycoplasma Pneumoniae-Induced Inflammation in Human Asthmatic Airway Epithelial Cells. J Immunol (2012) 188(7):3371–81. 10.4049/jimmunol.1100573.I. to T Kwon HY, Zimdahl. PMC3880785 22371396

[25] Li ZY Yang L Liu XJ Wang XZ Pan YX Luo JM. The Long Noncoding RNA MEG3 and Its Target Mir-147 Regulate Jak/Stat Pathway in Advanced Chronic Myeloid Leukemia. EBioMedicine (2018) 34:61–75. 10.1016/j.ebiom.2018.07.013 30072211 PMC6117736

[26] Li Z Yang L Liu X Nie Z Luo J. Long Noncoding RNA MEG3 Inhibits Proliferation of Chronic Myeloid Leukemia Cells by Sponging MicroRNA21. BioMed Pharmacother (2018) 104:181–92. 10.1016/j.biopha.2018.05.047 29772439

[27] Nie ZY Yao M Yang Z Yang L Liu XJ Yu J. De-Regulated STAT5A/miR-202-5p/USP15/Caspase-6 Regulatory Axis Suppresses CML Cell Apoptosis and Contributes to Imatinib Resistance. J Exp Clin Cancer Res (2020) 39(1):17. 10.1186/s13046-019-1502-7 31952546 PMC6969434

[28] Kang D Lee Y Lee JS. Rna-Binding Proteins in Cancer: Functional and Therapeutic Perspectives. Cancers (Basel) (2020) 12(9):2699. 10.3390/cancers12092699 PMC7563379 32967226

[29] Licatalosi DD Darnell RB. RNA Processing and its Regulation: Global Insights Into Biological Networks. %J Nature Reviews Genetics. Nat Rev Genet (2010) 11(1):75–87. 10.1038/nrg2673 20019688 PMC3229837

[30] Ge Y Schuster MB Pundhir S Rapin N Bagger FO Sidiropoulos N. The Splicing Factor RBM25 Controls MYC Activity in Acute Myeloid Leukemia. Nat Commun (2019) 10(1):172. 10.1038/s41467-018-08076-y 30635567 PMC6329799

[31] Gallardo M Lee HJ Zhang X Bueso-Ramos C Pageon LR McArthur M. Hnrnp K Is a Haploinsufficient Tumor Suppressor That Regulates Proliferation and Differentiation Programs in Hematologic Malignancies. Cancer Cell (2015) 28(4):486–99. 10.1016/j.ccell.2015.09.001 PMC4652598 26412324

[32] Ji D Zhang P Ma W Fei Y Xue W Wang Y. Oncogenic Heterogeneous Nuclear Ribonucleoprotein D-like Modulates the Growth and Imatinib Response of Human Chronic Myeloid Leukemia CD34 Cells Via Pre-B-cell Leukemia Homeobox 1. Oncogene (2020) 39(2):443–53. 10.1038/s41388-019-0998-9 31488872

[33] Liang Y Tebaldi T Rejeski K Joshi P Stefani G Taylor A. SRSF2 Mutations Drive Oncogenesis by Activating a Global Program of Aberrant Alternative Splicing in Hematopoietic Cells. Leukemia (2018) 32(12):2659–71. 10.1038/s41375-018-0152-710.1038/s41375-018-0152-7 PMC6274620 29858584

[34] Eiring AM Harb JG Neviani P Garton C Oaks JJ Spizzo R. miR-328 Functions as an RNA Decoy to Modulate Hnrnp E2 Regulation of mRNA Translation in Leukemic Blasts. Cell (2010) 140(5):652–65. 10.1016/j.cell.2010.01.007 PMC2924756 20211135

[35] Du Q Wang L Zhu H Zhang S Xu L Zheng W. The Role of Heterogeneous Nuclear Ribonucleoprotein K in the Progression of Chronic Myeloid Leukemia. Med Oncol (2010) 27(3):673–9. 10.1007/s12032-009-9267-z 19653139

[36] Chang JS Santhanam R Trotta R Neviani P Eiring AM Briercheck E. High Levels of the BCR/ABL Oncoprotein Are Required for the MAPK-hnRNP-E2 Dependent Suppression of C/EBPalpha-driven Myeloid Differentiation. Blood (2007) 110(3):994–1003. 10.1182/blood-2007-03-078303 17475908 PMC1924762

[37] Brandimarte L Pierini V Di Giacomo D Borga C Nozza F Gorello P. New MLLT10 Gene Recombinations in Pediatric T-acute Lymphoblastic Leukemia. Blood (2013) 121(25):5064–7. 10.1182/blood-2013-02-487256 23673860

[38] Yamazaki T Liu L Conlon E Manley JL. TCF3Burkitt Lymphoma-Related Mutations Alter TCF3 Alternative Splicing by Disrupting hnRNPH1 Binding. RNA Biol (2020) 17(10):1383–90. 10.1080/15476286.2020.1772559 PMC7549684 32449435

[39] Yamazaki T Liu L Manley JLJR. Tcf3 Mutually Exclusive Alternative Splicing Is Controlled by Long Range Cooperative Actions Between hnRNPH1 and PTBP1. RNA (2019) 25(11):1497–508. 10.1261/rna.072298.119 PMC6795145 31391218

[40] Amin HM Hoshino K Yang H Lin Q Lai R Garcia-Manero G. Pathology, Decreased Expression Level of SH2 Domain-Containing Protein Tyrosine Phosphatase-1 (Shp1) Is Associated With Progression of Chronic Myeloid Leukaemia. J Pathol (2007) 212(4):402–10. 10.1002/path.2178 17503411

[41] Huelga SC Vu AQ Arnold JD Liang TY Liu PP Yan BY. Integrative Genome-Wide Analysis Reveals Cooperative Regulation of Alternative Splicing by hnRNP Proteins. Cell Rep (2012) 1(2):167–78. 10.1016/j.celrep.2012.02.001 PMC3345519 22574288

[42] Lan X Zhao C Chen X Zhang P Zang D Wu J. Nickel Pyrithione Induces Apoptosis in Chronic Myeloid Leukemia Cells Resistant to Imatinib Via Both Bcr/Abl-dependent and Bcr/Abl-independent Mechanisms. J Hematol Oncol (2016) 9(1):129. 10.1186/s13045-016-0359-x 27884201 PMC5123219

[43] Tao T Yang X Zheng J Feng D Qin Q Shi X. PDZK1 Inhibits the Development and Progression of Renal Cell Carcinoma by Suppression of SHP-1 Phosphorylation. Oncogene (2017) 36(44):6119–31. 10.1038/onc.2017.199 28692056

[44] Jiang LC Luo JM. Role and Mechanism of Decitabine Combined With Tyrosine Kinase Inhibitors in Advanced Chronic Myeloid Leukemia Cells. Oncol Lett (2017) 14(2):1295–302. 10.3892/ol.2017.6318 PMC5529866 28789344

[45] Gagné JP Hunter JM Labrecque B Chabot B Poirier GG. A Proteomic Approach to the Identification of Heterogeneous Nuclear Ribonucleoproteins as a New Family of Poly(ADP-Ribose)-Binding Proteins. Biochem J (2003) 371(Pt 2):331–40. 10.1042/BJ20021675 PMC1223283 12517304

[46] Harrision D Gravells P Thompson R Bryant HE. Poly(Adp-Ribose) Glycohydrolase (PARG) *vs.* Poly(ADP-Ribose) Polymerase (Parp) - Function in Genome Maintenance and Relevance of Inhibitors for Anti-Cancer Therapy. Front Mol Biosci (2020) 7:191. 10.3389/fmolb.2020.00191 33005627 PMC7485115

